# Genome-Wide Identification and Characterization of the Growth-Regulating Factor Gene Family Responsive to Abiotic Stresses and Phytohormone Treatments in *Populus ussuriensis*

**DOI:** 10.3390/ijms26073288

**Published:** 2025-04-01

**Authors:** Ying Zhao, Yuqi Liu, Yuan Chai, Hedan Zhang, Ming Wei, Chenghao Li

**Affiliations:** 1 State Key Laboratory of Tree Genetics and Breeding, Northeast Forestry University, Harbin 150040, China; zhaoxxxy1@163.com (Y.Z.); liuyq00617@163.com (Y.L.); 18743581536@163.com (Y.C.); 18830228312@163.com (H.Z.); 2 School of Forestry, Northeast Forestry University, Harbin 150040, China

**Keywords:** growth-regulating factor, genome-wide analysis, abiotic stress, hormone treatment, protein–protein interaction, *Populus ussuriensis*

## Abstract

As a unique class of plant-specific transcription factors, the GROWTH-REGULATING FACTORs (GRFs) play pivotal roles in regulating plant growth, development, and stress responses. In this study, the woody plant *Populus ussuriensis* was taken as the research object. Nineteen *PuGRFs* were identified and classified into six clades, and their potential evolutionary relationships were analyzed. The possible biological functions of *PuGRFs* were speculated through bioinformatics analysis. Combining real-time fluorescence quantitative PCR, *PuGRFs* were determined to be actively expressed in young tissues, and there are distinct tissue-specific expressions in the mature tissues of woody plants. We also conducted RT-qPCR of PuGRFs under different abiotic stresses and phytohormone treatments, most of the family members were induced under the treatments of methyl jasmonate (MEJA) and salicylic acid (SA), and we also found that 4 of 19 PuGRFs might participate in abscisic acid (ABA)-mediated osmotic stress in roots. Protein–protein interaction prediction analysis showed that six *PuGRFs* can interact with two types of growth-regulating interaction factors (GIFs). Further prediction and verification revealed that *PuGRF1/2c* and *PuGRF1/2d*, which belong to the same clade and have highly similar sequences, exhibited divergent interaction capabilities with GIFs, indicating evolutionary fine-tuning and functional redundancy within the GRF family. These findings lay a foundation for studying the molecular mechanisms of *PuGRFs* in *P. ussuriensis*, suggest that *PuGRFs* play important roles in responding to hormones and environmental changes, and the potential interaction relationships are worthy of exploration.

## 1. Introduction

The growth and development of plants is an exceedingly complex process delicately regulated by multiple genes [1]. GROWTH-REGULATING FACTOR (GRF) belongs to a category of transcription factors unique to plants, and plays an extremely crucial role in the process of plant growth and development. *OsGRF1* was initially discovered and named in rice (*Oryza sativa*) [2]. Shortly after that, more GRFs were successively identified in various plants. In herbaceous plants like *Arabidopsis thaliana*, *O. sativa*, and *Zea mays*, a total of 9, 12, and 14 *GRF* family members have been successfully identified respectively. Similarly, in woody species such as *Populus trichocarpa* [3], *Cajanus cajan* [4], *Jatropha curcas* [5], and *Citrus sinensis* [6], 19, 10, 10, and 19 *GRF* members have been detected. The identification of members of the *GRF* gene family mainly relies on unique structural characteristics [7]. The N-terminal region harbors two highly conserved domains: QLQ (glutamine, leucine, and glutamine) and WRC (tryptophan, arginine, and cysteine). The QLQ domain interacts with the SNH domain of growth-regulating interaction factors (GIFs). GIFs function by forming GRF–GIF complexes, which play a regulatory role in processes like cell proliferation, differentiation, and organ formation [8,9,10]. The WRC domain exhibits even greater sequence conservation, encompassing a nuclear localization signal and a C3H-type zinc finger motif. This domain can be tightly bound to the cis-acting elements of downstream genes to regulate the temporal and spatial expression of genes. In contrast, the C-terminal sequence of the GRF protein has a wide variation range. Some motifs with relatively low conservation, such as TQL, GGPL, and FFD, typically appear at the C-terminal [11,12,13]. The conserved and diverse characteristics of the plant GRF domains collectively endow GRFs with the ability to play crucial functions in all aspects of plant growth and development.

In plants, GRFs were shown significantly to contribute to the early growth and development of plants and play crucial regulatory roles in the formation of plant tissues and organs. The *GRF* gene family in *A. thaliana* is widely involved in the growth process, such as *AtGRF1*, which regulates leaf size and cell proliferation; *AtGRF2*, involved in controlling root growth and plant height [7]; and *AtGRF5*, which promotes leaf growth by influencing cell division in leaf primordia [14]. Previous studies have shown that *OsGRF4* regulates grain size and yield by modulating cell division and expansion in developing seeds [15]. *OsGRF7* and *OsGRF8*, together with GA, were shown to determine rice leaf length by regulating cell division zone size [16]. In addition, *OsGRF1* is shown to influence rice leaf growth [2].

Plants often face multiple stressors in nature, and transcription factors (TFs) play a crucial role in regulating stress-responsive genes to help plants adapt to adverse environmental conditions. Previous studies have shown that the *AtGRF7*-targeted cis-element (TGTCAGG) negatively regulates multiple downstream responses to osmotic stress in *A. thaliana* [17]. *PdbGRF1* banded to the drought response element (DRE, ‘A/GCCGAC’) to regulate the expression of *PdbPOD17* and *PdbAKT1* and activate the related physiological pathways, showing a positive regulatory effect on salt stress in *Populus davidiana* × *P. bolleana* [18]. In addition to directly regulating other genes as transcription factors, GRFs can also interact with other proteins. For example, the *A. thaliana* AtGRF–AtGIF complex binds to promoters of cell cycle genes, like CYCLIN family members, during early leaf development. Multiple AtGRFs interacted with DELLA and regulated plant responses to cold stress [19]. It has been reported in studies that all 19 *GRFs* in poplar are predicted to be regulated by miRNA396 [20,21,22], and they are mainly recognized in the region encoding the last few amino acids of the WRC domain. Although there are multiple reports in the literature on the functions of *GRFs* in improving the early growth and development of woody plants, the relevant research on woody plants, to a certain degree, trailed behind that in herbaceous plants.

*P. ussuriensis*, one of the main fast-growing tree species in the mountainous forest regeneration of eastern Northeast China, is early maturity, high-yield, and has high economic value. In this research, we leveraged *P. ussuriensis* materials to conduct a more in-depth exploration of the evolutionary dynamics of GRFs in woody plants via bioinformatics analysis. We detected the expression profiles of *PuGRFs* across various tissues during the seedling and rapid-growth stages. Our findings revealed that, as the plant transitioned through different growth and development phases, the tissue-specificity of *PuGRFs* underwent a notable shift. By simulating the environmental challenges that trees may encounter through abiotic stress and phytohormone treatments, we observed that the majority of *PuGRFs* displayed similar expression patterns in leaves in response to phytohormone treatments. Moreover, a subset of *PuGRFs* potentially responded to drought or salt stress mediated by ABA. Concurrently, we employed predictive models to forecast the potential interaction relationships among PuGRFs. Subsequently, we selected two highly representative genes to conduct preliminary verification of their interaction with PuGIFs. The outcomes of this study not only present a comprehensive panorama of the PuGRF gene family, but also open up novel perspectives for the further exploration and validation of the functions of PuGRF genes in the woody plant *P. ussuriensis*.

## 2. Results

### 2.1. Genome-Wide Discovery of GRFs in P. ussuriensis

To identify the *GRF* gene family in the *P. ussuriensis* genome, we used transcriptome sequencing data of *P. ussuriensis* obtained from the previous work of our laboratory. Since the genomes of *P. ussuriensis* and *P. trichocarpa* are very similar [23,24,25] (Figure S1), we screened 19 genes of the *GRF* gene family in *P. ussuriensis* based on that of *P. trichocarpa*. We successfully pinpointed 19 members of the *GRF* gene family in *P. ussuriensis*. These genes were designated *PuGRF1/2a toPuGRF12b*, primarily based on their homology with *PtGRFs*.

Subsequently, basic information on these identified *GRFs* was compiled (Table 1). Nineteen *PtGRFs* were unevenly distributed across eleven chromosomes. The open-reading frame (ORF) of the 19 *PuGRFs* represents CDS ranging in length from the shortest 606 bp (*PuGRF12b*) to the longest 1836 bp (*PuGRF1/2d*), which encode proteins with lengths ranging from 201 to 611 amino acids (aa), molecular weight (Mw) ranging from 22.32 kDa to 66.53 kDa, and isoelectric points (pI) ranging between 6.44 and 10.06. Except for *PuGRF12b*, which may be localized in the mitochondrion and nucleus, the predicted localization of other *PuGRFs* is in the nucleus. Predicting microRNAs shows that all *PuGRFs* could be recognized and cleaved by ptc-miR396a to ptc-miR396g.

### 2.2. Protein Evolution of PuGRFs

**Figure 1:**
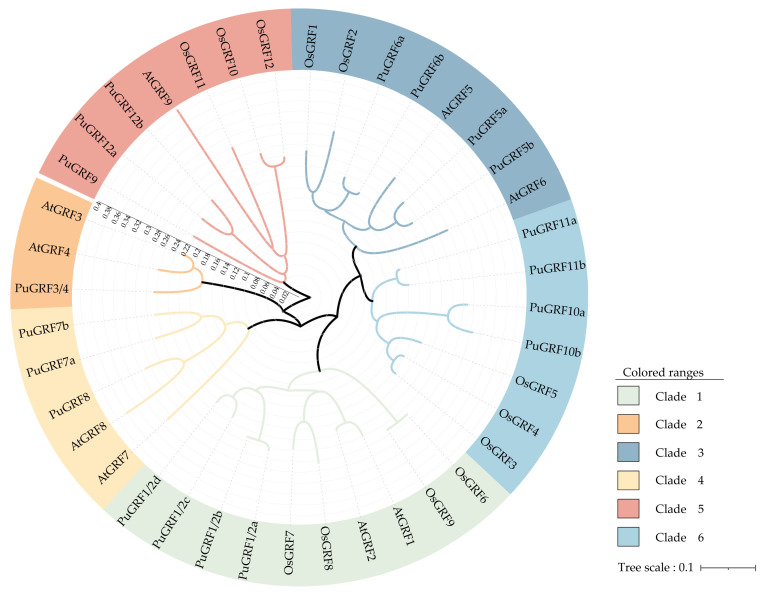
Phylogenetic analysis of GRF proteins across *P. ussuriensis*, *O. sativa*, and *A. thaliana*.

To uncover the phylogenetic relationship, an interspecific phylogenetic tree was constructed using 40 *GRFs* from *P. ussuriensis* (19), *O. sativa* (12), and *A. thaliana* (9) (Figure 1). The results demonstrated that the *PuGRFs* were divided into six clades. Meanwhile, the distribution of *GRFs* in poplar, arabidopsis, and rice was different. *OsGRFs* were only distributed in clades I, III, V, and VI. *AtGRFs* were not distributed in clade VI. The obvious differences in the interspecific phylogenetic tree indicate that the evolutionary history of *GRFs* is rich and complex, potentially reflecting functional diversification and adaptation to different ecological niches.

Phylogenetic analysis was performed using MEGA7.0 software with the adjacency (NJ) method and 1000 iterations were carried out for bootstrap testing. The modules are color-coded to represent the six clades of *GRFs*. The 9 members in *A. thaliana* and 12 members in *O. sativa* are as follows: AT2G22840.1, AT4G37740.1, AT2G36400.1, AT3G52910.1, AT3G13960.1, AT2G06200.1, AT5G53660.1, AT4G24150.1, AT4G24150.1, LOC_Os02g53690.1, LOC_Os06g10310.1, LOC_Os04g51190.1, LOC_Os02g47280.1, LOC_Os06g02560.3, LOC_Os03g51970.1, LOC_Os12g29980.1, LOC_Os11g35030.1, LOC_Os03g47140.1, LOC_Os02g45570.1, LOC_Os07g28430.1, and LOC_Os04g48510.1.

### 2.3. The Protein Motif, Structure, and Cis-Element Analysis of PuGRFs

**Figure 2:**
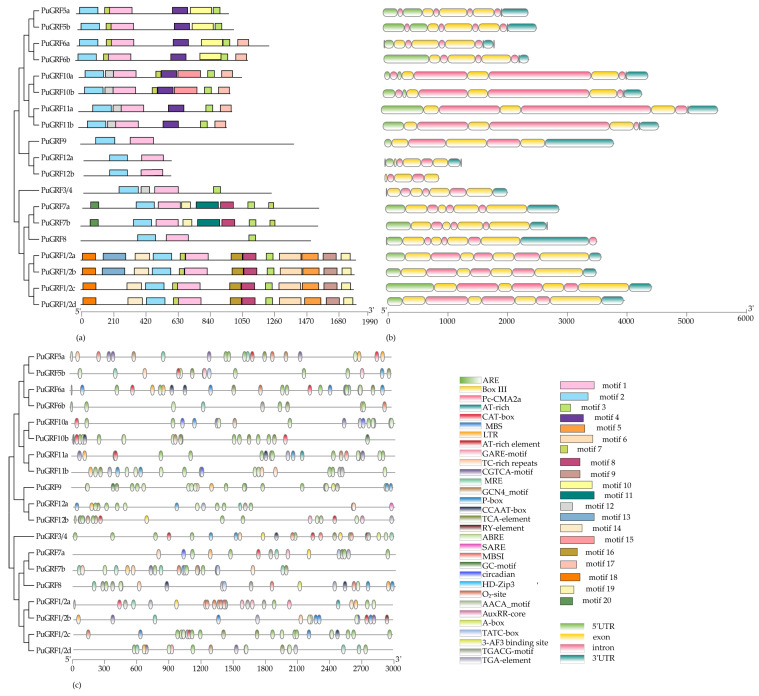
Phylogenetic tree and structure of PuGRFs. (**a**) Conserved motifs identified in PuGRF proteins using MEME. Twenty motifs (1–20) are color-coded, with the QLQ domain (blue) and WRC domain (pink) universally conserved. (**b**) The gene structure of the *PuGRF* genes. Yellow and pink boxes represent exons and introns, respectively; blue and green boxes denote 3′UTR and 5′UTR regions. Gene lengths (base pairs) are scaled proportionally. (**c**) Distribution of cis-regulatory elements in the 3.0 kb promoter regions of *PuGRFs*. The boxes in different colors respectively represent 31 types of cis-elements.

To understand the unification and diversification of the *PuGRFs*, analyses of conserved motifs, gene structure, and cis-elements were conducted. Protein motifs are not only key sites for the mutual recognition and binding between proteins, but also capable of mediating the interactions between proteins and nucleic acids. Regarding protein motifs, motif 1 and motif 2, annotated as the *GRF* domain, were identified in all clades (Table S4). Except for clade II, where only *PuGRF3/4* was present, and clade IV, where there was a relatively large difference among *PuGRF7a*, *PuGRF7b*, and *PuGRF8*, the *PuGRFs* belonging to the same clade showed high agreement in motifs (Figure 2a).

The gene structure reflects the evolutionary history of genes to a certain extent. During the evolutionary process, the structures of some key genes often show a high degree of conservation. The 19 *PuGRFs* identified in this study range in length from 914 to 5642 base pairs, each containing intron structures and either 3 or 4 exons (Figure 2b). Although there is little difference in the number of structures, *PuGRF10a-PuGRF11b*, which belong to clade VI, encode medium-length CDS with a longer intron. Notably, *PuGRF12b* lacks the 5′ UTR and 3′ UTR regions, while the remaining genes possess the typical 5′ UTR and 3′ UTR structures.

The 3.0 kb promoter region of all *PuGRFs* was utilized to accurately predict the promoter *cis*-elements and explore the possible biological roles of *PuGRFs* (Figure 2c). In addition to TATA-box, CAAT-box, and GC-box, which are essential for initiating transcription, a total of 31 *cis*-regulatory elements were obtained and classified into the following categories according to their functions (Table S5): (1) Binding sites for proteins or specific transcription factors, such as CCAAT-boc (MYBHv1 binding site) and HD zip 3 (proteins binding site); (2) Photoreactive elements. Each *GRF* promoter region contains a large number of photoreactive elements, such as MRE and Pc-CMA2a. (3) Growth and development response elements. These include CAT (meristematic tissue), RY-element (seed specificity), GCN4-motif (endosperm expression), and circadian; (4) Hormone response elements. These include TGA (methyl jasmonate), ABRE (abscisic acid), GARE (gibberellin), and TCA-element (salicylic acid); and (5) Environmental stress response elements, such as TC-rich (defense and stress response), LTR (cold), ARE (anaerobic induction), and MBS (drought).

The results indicate that the number of promoter elements in the entire family ranges from 7 to 15, with little interspecies variation. For instance, with the exception of *PuGRF7a*, all *GRFs* possess elements that respond to abscisic acid. There are 12 *GRFs* with cis-acting elements involved in defense and stress responses, while the other 7 *GRFs* lack this element, but have elements that respond to low temperatures or droughts. Collectively, these findings strongly suggest that *GRF* genes may indeed be involved in biological functions related to these hormone and stress response pathways [26].

### 2.4. Expression Pattern of PuGRFs

#### 2.4.1. Expression Analysis of PuGRFs During Growth and Development of *P. ussuriensis*

We selected various tissues from two growth stages of *P. ussuriensis*: the seedling stage (young root (YR), young stem (YS), young leaves (YLs), and bud)), and the rapid growth stage (mature root (MR), phloem, cambium, xylem, mature leaves (MLs), and apical bud (AP)) to examine the expression of *PuGRFs*. By analyzing expression changes in various tissues of woody plants across different growth stages, this study suggests that the functions of *PuGRFs* exhibit potential conservation and divergence compared with those in herbaceous plants.

**Figure 3:**
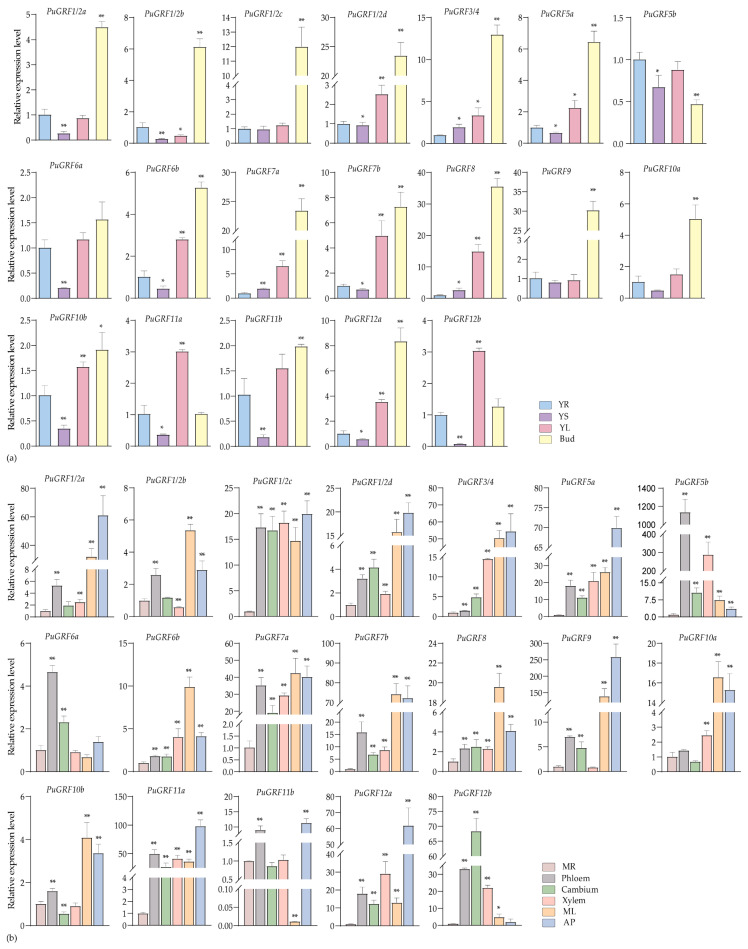
Tissue-specific expression profiles of *PuGRFs* during seedling and rapid growth stages. (**a**) The expression of *PuGRFs* at the seedling stage. The different tissues are respectively YR (young root), YS (young stem), YLs (young leaves), and Bud. (**b**) The expression of *PuGRFs* at the rapid growth stage. The different tissues are respectively MR (mature root), phloem, cambium, xylem, MLs (mature leaves), and AP (apical bud). The different color blocks represent the different tissues. The standard deviation is shown at the top of the bar graph. Data represent the mean ± SD (*n* = 3). Asterisks denote significant differences (* *p* < 0.05, ** *p* < 0.01, Student’s *t*-test).

The results showed that, in the materials at the seedling stage, most of the *PuGRFs* were regularly highly expressed in young leaves (YL) and bud tissues, with the lowest expression levels in YS, except for *PuGRF5b* (Figure 3a). It should be particularly noted that the expression levels of *PuGRF1/2d*, *PuGRF7a*, *PuGRF8*, and *PuGRF9* in buds were more than 20 times higher than those in YR, YS, and YL during the same period. During the rapid growth stage, *PuGRFs* exhibit diverse tissue specificity. Twelve *PuGRFs* were specifically highly expressed in ML and AP. Furthermore, relatively high expression levels of *PuGRF1/2c*, *PuGRF7a*, *PuGRF11a*, *PuGRF12a*, and *PuGRF12b* were observed in phloem, cambium, and xylem tissues. This result is likely due to the involvement of these *PuGRFs* in stem differentiation changes associated with woody plant growth cycle transitions, hinting at their potential roles in radial development, particularly for the paralogous pair *PuGRF12a* and *PuGRF12b*. Among them, *PuGRF1/2c* and *PuGRF11a* have different expression patterns from their homologous gene pairs *PuGRF1/2d* and *PuGRF11b*, respectively. *PuGRF1/2d* is significantly expressed in ML and AP, and *PuGRF11b* is significantly expressed in phloem and AP, suggesting functional differences among homologous genes (Figure 3b).

#### 2.4.2. Expression Analysis of PuGRFs Under Salt and Osmotic Stresses

We analyzed the expression patterns of *PuGRFs* in *P. ussuriensis* under salt and osmotic stress during the seedling stage at different time points (0 h, 3 h, 6 h, 12 h, 24 h, 48 h, 72 h, and 7 days).

**Figure 4:**
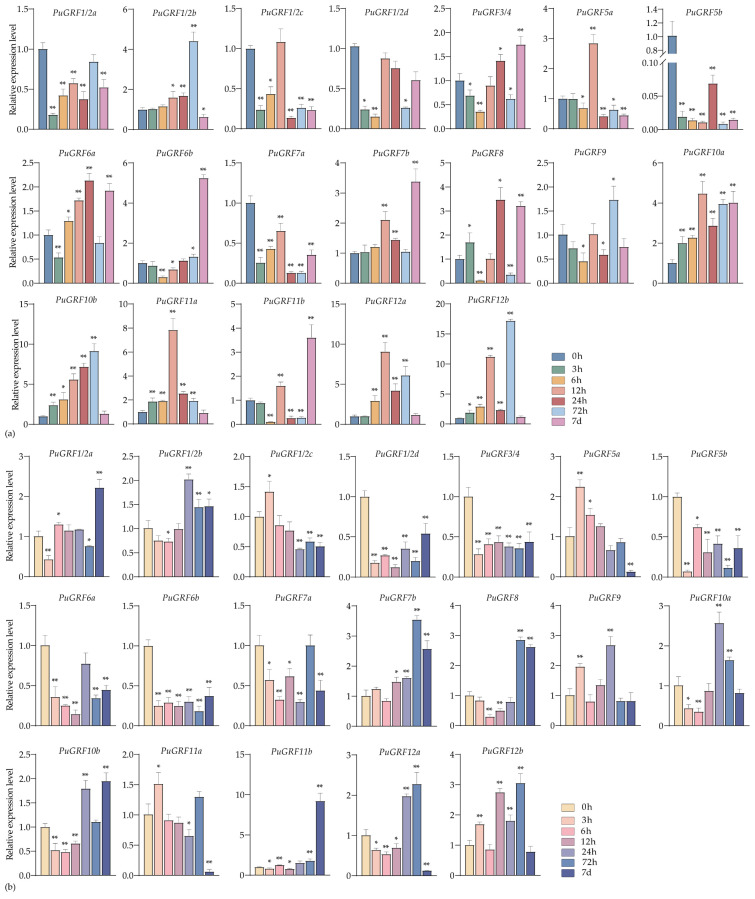
Expression patterns of *PuGRFs* under 200 mM NaCl stress. Different colored blocks represent the samples collected at 0 h, 3 h, 6 h, 12 h, 24 h, 48 h, 72 h, and 7 days after the stress. (**a**,**b**) respectively show the expressions of *PuGRFs* in the leaves and roots of *P. ussuriensis*. Standard deviation is shown at the top of the bar graph. Data represent the mean ± SD (*n* = 3). Asterisks denote significant differences (* *p* < 0.05, ** *p* < 0.01, Student’s *t*-test).

Under salt stress of 200 mM NaCl, in leaves, eight *PuGRFs* showed a rapid up-regulation response and continued to be induced (Figure 4a). In roots, although a few *PuGRFs* showed rapid up-regulation in response to the salt stress, six *PuGRFs* could function under salt stress at 7 days (Figure 4b). The results showed that *PuGRF7b*, *PuGRF8*, and *PuGRF11b* were induced in both leaves and roots under salt stress at 7 days. We speculate that these genes are helpful for *P. ussuriensis* to adapt to the salt stress environment.

**Figure 5:**
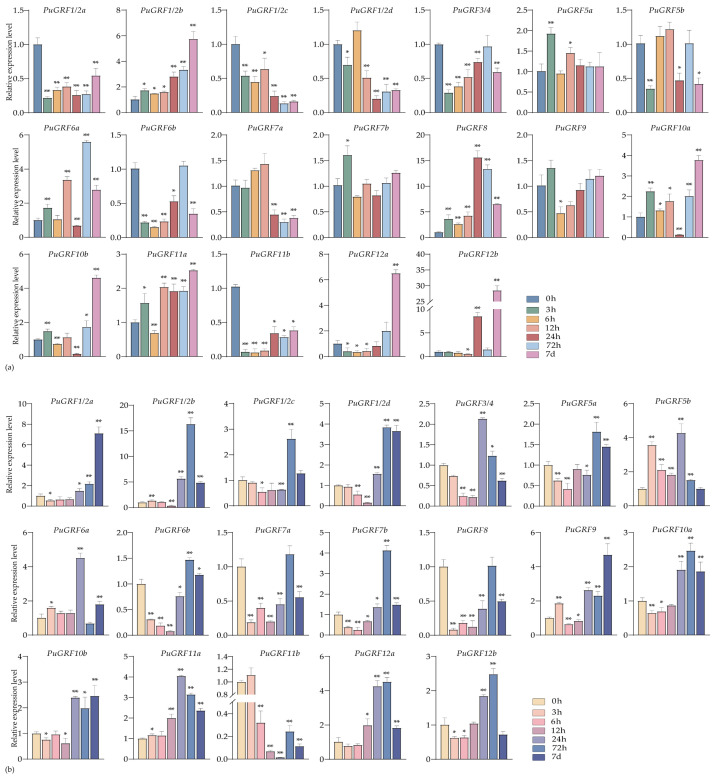
Expression patterns of *PuGRFs* under osmotic stress. Different colored blocks represent the samples collected at 0 h, 3 h, 6 h, 12 h, 24 h, 48 h, 72 h, and 7 days after the stress. (**a**,**b**) respectively show the expressions of *PuGRFs* in the leaves and roots of *P. ussuriensis*. Standard deviation is shown at the top of the bar graph. Data represent the mean ± SD (*n* = 3). Asterisks denote significant differences (* *p* < 0.05, ** *p* < 0.01, Student’s *t*-test).

Under the osmotic stress induced by 7% PEG6000, the expression of nine *PuGRFs* was up-regulated in both leaves and roots at 7 days (Figure 5a,b). We found that *PuGRF1/2b* and *PuGRF8* were consistently up-regulated in leaves, maintaining an active response throughout the stress process at various time points. In roots, most *PuGRFs* exhibited an expression pattern of being first inhibited by stress and then induced, except for *PuGRF1/2b*, *PuGRF5b*, *PuGRF6a*, *PuGRF9*, *PuGRF11a*, *PuGRF11b.* Among them, *PuGRF1/2c*, *PuGRF1/2d*, *PuGRF5a*, *PuGRF6b*, *PuGRF7b*, *PuGRF10a*, *PuGRF12a*, and *PuGRF12b* all reached their highest expression levels at 72 h. These research findings may establish a potential link between *PuGRFs* and complex drought-response mechanisms.

#### 2.4.3. Expression Analysis of PuGRFs Under ABA, MeJA and SA Treatment

We also analyzed the expression patterns of *PuGRFs* under ABA, MeJA, and SA treatment during the seedling stage at different time points (0 h, 3 h, 6 h, 12 h, 24 h, 48 h, 72 h, and 7 days).

**Figure 6:**
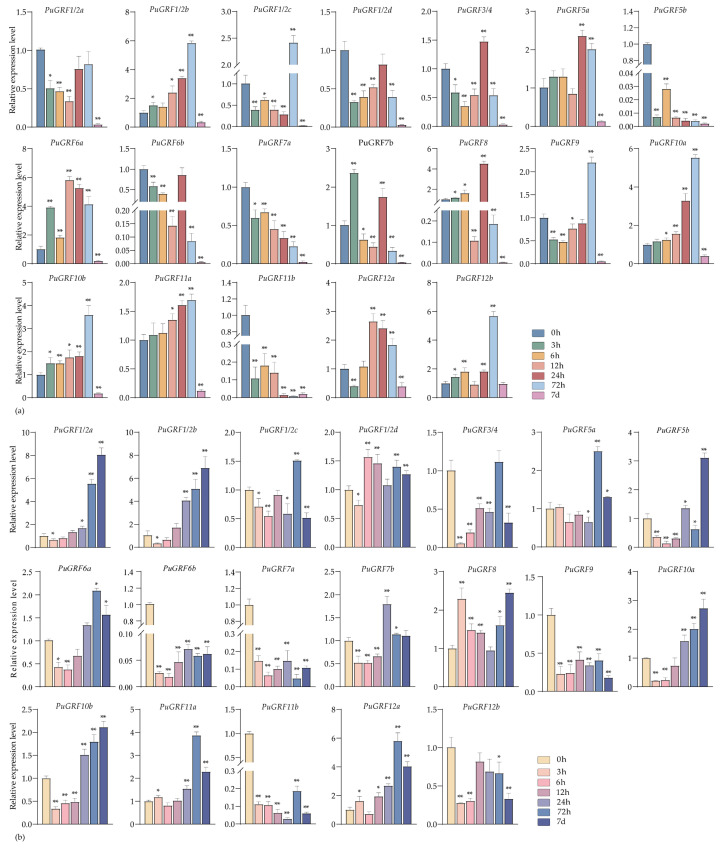
Expression patterns of *PuGRFs* under the treatment of 200 μM ABA. Different color blocks represent the samples collected at 0 h, 3 h, 6 h, 12 h, 24 h, 48 h, 72 h, and 7 d after the stress. (**a**,**b**) respectively show the expressions of *PuGRFs* in leaves and roots of *P. ussuriensis*. The standard deviation is shown at the top of the bar graph. Data represent the mean ± SD (*n* = 3). Asterisks denote significant differences (* *p* < 0.05, ** *p* < 0.01, Student’s *t*-test).

Under the treatment of 200 μM ABA, in leaves, upon reaching a treatment duration of 72 h, the expression levels of seven genes attained their peak values. Under the treatment of ABA at 7 days, the expression levels of all *PuGRFs* diminished to the lowest point (Figure 6a). In roots, the expression of 12 *PuGRFs* was induced and up-regulated by 7 days (Figure 6b). Meanwhile, the homologous genes *PuGRF1/2a* and *PuGRF1/2b* had a consistent expression pattern in roots and exhibited an almost opposite expression pattern in leaves. This phenomenon prompts us to postulate the existence of functional redundancy and tissue-specificity under the treatment of ABA.

**Figure 7:**
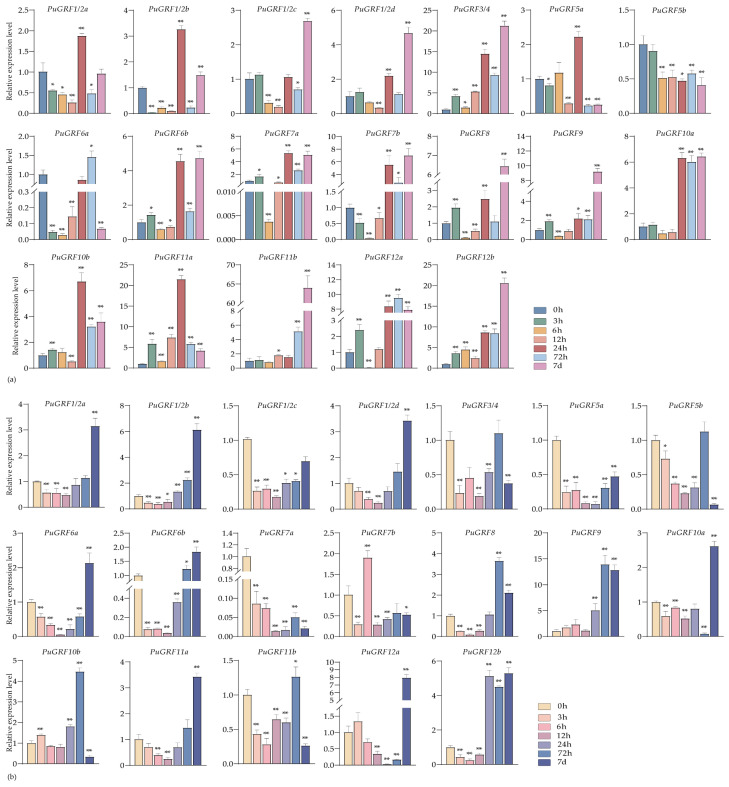
Expression patterns of *PuGRFs* under the treatment of 100 μM MeJA. Different color blocks represent the samples collected at 0 h, 3 h, 6 h, 12 h, 24 h, 48 h, 72 h, and 7 days after the stress. (**a**,**b**) respectively show the expressions of *PuGRFs* in the leaves and roots of *P. ussuriensis*. The standard deviation is shown at the top of the bar graph. Data represent the mean ± SD (*n* = 3). Asterisks denote significant differences (* *p* < 0.05, ** *p* < 0.01, Student’s *t*-test).

Under the treatment of 100 μM MeJA, the expression of 10 *PuGRFs* was induced in both leaves and roots at 7 days (Figure 7a,b). As the results indicate, *PuGRFs* without MeJA-binding elements, such as *PuGRF5b*, are sensitive to MeJA. However, *PuGRFs* like *PuGRF12b* show continuous and significant induced expression in leaves and are also induced in roots during the treatment period from 24 h to 7 days. These results confirm that MeJA treatment can indeed induce the expression of *PuGRFs*.

**Figure 8:**
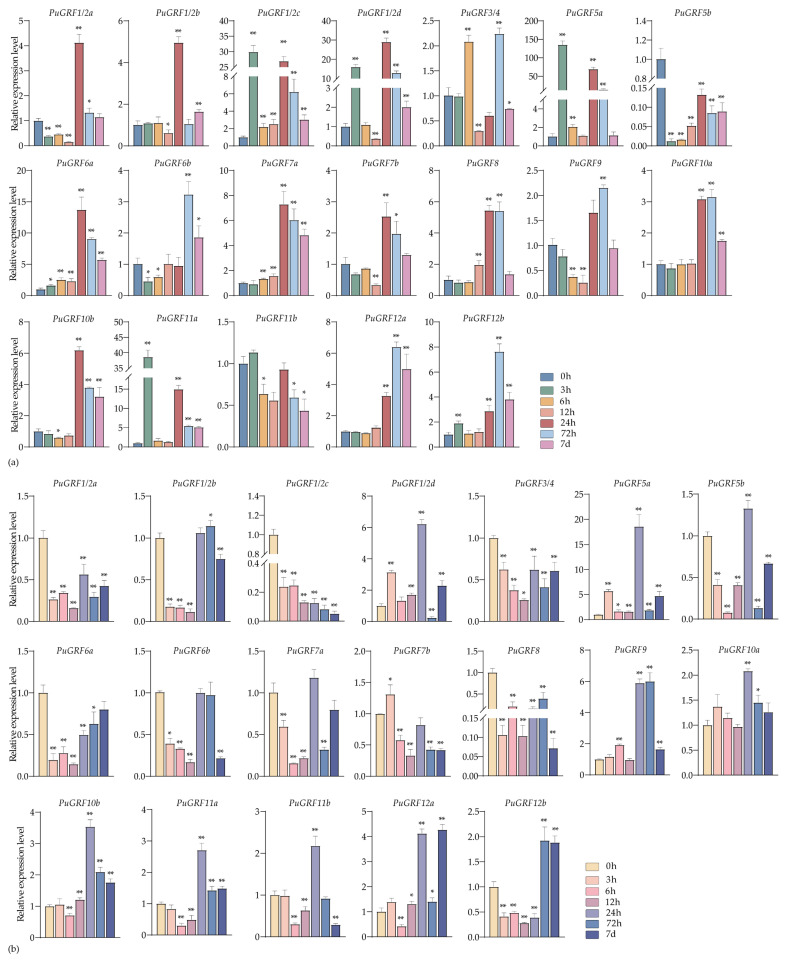
Expression patterns of *PuGRFs* under the treatment of 100 μM SA. Different color blocks represent the samples collected at 0 h, 3 h, 6 h, 12 h, 24 h, 48 h, 72 h, and 7 days after the stress. (**a**,**b**) respectively show the expressions of *PuGRFs* in leaves and roots of *P. ussuriensis*. The standard deviation is shown at the top of the bar graph. Data represent the mean ± SD (*n* = 3). Asterisks denote significant differences (* *p* < 0.05, ** *p* < 0.01, Student’s *t*-test).

Under the treatment of 100 μM SA, in leaves, only *PuGRF5b* and *PuGRF11b* showed obvious sensitivity, and the remaining *PuGRFs* responded to varying degrees, especially with obvious time-specificity at 24 h and 7 days (Figure 8a). In roots, 11 *PuGRFs* exhibited an expression pattern of being first inhibited and then induced. Among them, except for *PuGRF1/2c* and *PuGRF5a*, all *PuGRFs* started to resume responding and were up-regulated at 24 h (Figure 8b). We speculate that the key time for *PuGRFs* to respond to SA may start from 24 h. All these results indicate that *PuGRFs* may be involved in the complex processes of hormone signal transduction and regulation, helping plants to better adapt to environmental stresses.

### 2.5. Predict and Analyze the Interacting Proteins of PuGRFs

**Figure 9:**
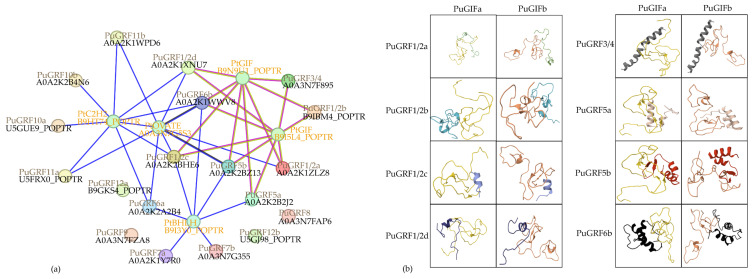
Predicted protein interaction networks and structural models of PuGRFs. (**a**) STRING-based PPI network of PtGRFs (black), their *P. ussuriensis* homologs (brown), and predicted interacting proteins (orange). Interaction lines indicate co-expression (black), gene fusion (red), text mining (yellow), and co-occurrence (blue). (**b**) HDOCK-predicted binding models between PuGRFs (green, blue, purple, etc.) and PuGIFs (yellow: PuGIFa; brown: PuGIFb). Docking scores and confidence values (Table 2) reflect interaction likelihood.

Utilizing the STRING12.0 online tool, we found that the possible interaction relationships with PuGRFs can be divided into two categories. One category is the interaction with the GRF’s partner protein GIF. Specifically, PuGRF1/2a, PuGRF1/2b, PuGRF1/2c, PuGRF1/2d, PuGRF3/4, PuGRF5a, PuGRF5b, and PuGRF6b all have interactions with GIFs (B9I5L4 and B9N9U1). The other category is gene co-occurrence with genes possessing the ZnF-C2H2 domain (B9HT74), OVATE domain (A0A3N7G5S3), and BHLH domain (B9I3Y0) (Figure 9a). Through sequence alignment, we obtained the two most homologous *PuGIFs* in *P. ussuriensis*, which were named *PuGIFa* and *PuGIFb* respectively. The HDOCK server was used to simulate the protein–protein interaction models between the above-mentioned eight PuGRFs and PuGIFa/PuGIFb (Figure 9b). The scoring results showed that, except for the interaction between PuGRF1/2c and the two PuGIFs being unlikely, interactions between the other seven PuGRFs and PuGIFs were possible. Specifically, the likelihood of interaction was moderate between PuGRF1/2a, PuGRF1/2d, PuGRF5b, and the two PuGIFs, between PuGRF3/4 and PuGIFb, and between PuGRF5a and PuGIFa. Interaction was highly likely between PuGRF1/2b, PuGRF6b, and the two PuGIFs, between PuGRF3/4 and PuGIFa, and between PuGRF5a and PuGIFb (Table 2).

### 2.6. Verification of the Interaction Relationship Between PuGRFs and PuGIFa/b

**Figure 10:**
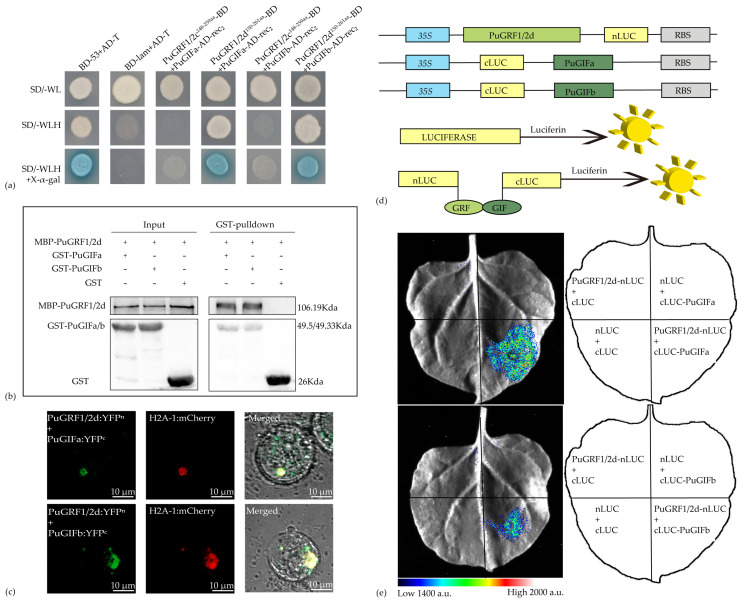
Experimental validation of PuGRF1/2d interactions with PuGIFa/b. (**a**) Y2H assays.t the interaction relationships between PuGRF1/2c, PuGRF1/2d, and PuGIFa or PuGIFb. PuGRF1/2c^148−259aa^ and PuGRF1/2d^150−261aa^ respectively represent the regions without activation activity of PuGRF1/2c and PuGRF1/2d (Figure S2). SD/-WL stands for SD/-Trp/-Leu. SD/-WLH stands for SD/-Trp/-Leu/-His. SD/-WLH + X-α-gal stands for SD/-Trp/-Leu/-His + X-α-gal. Controls: BD-53 + AD-T (positive), BD-lam + AD-T (negative). (**b**) In vitro pull-down assays. MBP-PuGRF1/2d interacted with GST-PuGIFa/b. The input served as the control group. Immunoblotting analysis was carried out using anti-GST and anti-MBP antibodies. (**c**) BiFC assays. PuGRF1/2d interacted with PuGIFa/b. PuGRF1/2d and PuGIFa or PuGIFb were respectively fused to the N-terminal and C-terminal of yellow fluorescent protein (YFP) and then co-transfected into protoplast cells. The nYFP and cYFP were set as controls. Scale bar = 10 µm (Figure S3). (**d**,**e**) LCI assays. PuGRF1/2d interacted with PuGIFa/b. Various constructs were co-infiltrated into the leaves of *N. benthamiana*, and the luciferase (LUC) activity was captured (as shown in the upper panel). The four squares in the lower part showed different combinations of constructs co-infiltrated into the leaves of *N. benthamiana*. The pseudocolor bar shows the range of luminescence intensity.

Based on the predicted protein interaction results, we selected PuGRF1/2c, PuGRF1/2d, PuGIFa, and PuGIFb for experimental validation. Domain mapping experiments conducted through yeast two-hybrid (Y2H) assays indicated that the QLQ and WRC domains of PuGRF1/2c and PuGRF1/2d were involved in the interaction between PuGRFs and PuGIFs (Figure S2). Consistent with the prediction results of the HDOCK SERVER, PuGRF1/2d interacted with PuGIFa/b, while PuGRF1/2c failed to interact with PuGIFa/b (Figure 10a). Next, in order to determine the accuracy of the interaction between PuGRF1/2d and PuGIFa/b, we further demonstrated, through in vitro pull-down assays, that PuGIFa/b tagged with glutathione S-transferase (GST) (GST-PuGIFa/b), rather than the GST protein itself, could pull down PuGRF1/2d tagged with maltose-binding protein (MBP) (MBP-PuGRF1/2d) (Figure 10b), thus confirming that PuGRF1/2d and PuGIFa/b interact with each other in vitro. Bimolecular fluorescent complementation (BiFC) also showed that yellow fluorescence could only be detected when PuGRF1/2d and PuGIFa/b were fused to the N-terminal and C-terminal of yellow fluorescent protein (YFP), respectively, and were present simultaneously (Figure 10c and Figure S3). This further confirmed that PuGRF1/2d and PuGIFa or PuGIFb interact with each other in vitro.

Subsequently, we utilized the well-established firefly luciferase (LUC) complementation imaging (LCI) assay in *Nicotiana benthamiana* leaves to determine whether PuGRF1/2d and PuGIFa/b interact with each other in planta. A strong fluorescence signal was observed in *N. benthamiana* cells co-expressing PuGRF1/2d-nLUC and cLUC-PuGIFa/b, whereas no signal was detected in those co-expressing PuGRF1/2d-nLUC and cLUC, nLUC, and cLUC-GIFs or nLUC and cLUC (Figure 10d,e). These results indicate that PuGRF1/2d and PuGIFa/b interact with each other in vivo.

## 3. Discussion

As key players in the regulatory network of plant growth and development, *GRFs* play a crucial role throughout the entire life cycle of plants. *GRFs* precisely regulate cell proliferation and differentiation, and actively participate in the process of plant responses to various environmental stresses, thereby helping plants to adjust their growth strategies and enhance their tolerance to adverse environments. Although certain progress has been made in the research on the GRF gene family in most herbaceous plants, studies on higher woody plants still lag behind. Therefore, we conducted a genome-wide identification and analysis of the GRF gene family in *P. ussuriensis*, one of the tree species with strong regional growth, and deeply explored its potential functions and action mechanisms.

We referred to the basic information of *GRFs* in *P. trichocarpa* and identified 19 *PuGRFs* to study their evolutionary relationships, gene structures, and conserved motifs. A phylogenetic tree was constructed with the *GRF* family members of *A. thaliana* and *O. sativa*. The results showed that *PuGRFs* were unevenly classified into six clades. *PuGRFs* belonging to the same clade had more similar gene structures and protein motifs. Consistent with this, numerous studies have found that genes within the same clade have similar functions. For instance, *OsGRF6*, *OsGRF7*, *OsGRF8*, and *OsGRF9* exhibit a strong response to *Magnaporthe oryzae* [27]. *AtABF2*, *AtABF3*, *AtABF4*, and *TaABF* are all key regulatory factors in the ABA signal transduction pathway [28,29]. In our study, different from the gene structures of *A. thaliana*, *O. sativa*, and *C. sinensis* [6,7,30], which have 2–4 exons, all *PuGRFs* have 4 exons, except for *PuGRF9*, *PuGRF12a*, and *PuGRF12b* in the clade V, which have only 3 exons. Compared with other genes in the same clade, the 4 *PuGRFs* in clade I have more than 10 protein motifs, especially showing significant diversity at the C-terminus. In addition, the evolutionary branches where most *PuGRFs* are located have experienced multiple evolutionary nodes. Overall, we speculate that these results are due to the diversification and adaptive changes that *PuGRFs* have undergone during the evolutionary process.

Plant morphogenesis and every growth stage rely on the tissue-specific expression of various genes. In this study, we selected various tissues from two critical growth stages of *P. ussuriensis* to analyze the tissue specificity of *PuGRFs*. The results showed that most *PuGRFs* were specifically expressed in the buds and YL at the seedling stage, as well as in the ML and AP at the rapid growth stage. This result is consistent with previous studies [11,16,31,32]. Furthermore, some *GRFs* are expressed in the root cap or meristem and have an important impact on root growth and branching, such as *MtGRF5* in *Medicago truncatula* and *PtGRF1/2d* [33,34]. This tissue specificity often also represents a degree of functional specificity; for example, the specific expression of *AtARF7* and *AtARF19* can regulate the formation of lateral roots and primary roots in *A. thaliana* [35]. The specific expression of *MtARF2*, *MtARF3*, and *MtARF4* in *M. truncatula* [36] is a crucial morphogenetic program that controls root system development and the formation of nitrogen-fixing nodules. Compared with herbaceous plants with a relatively short growth cycle, the functions of *GRFs* in woody plants are perhaps more differentiated in different tissues. For example, *PuGRF5b* was expressed at a higher level in the roots at the seedling stage, while at the rapid growth stage, it was predominantly expressed in the phloem. We speculated that the function of *PuGRF5b* in poplar may have evolved based on the potential regulatory effect of its homologous *OsGRF1* in response to gibberellin and on stem growth [30]. In addition, *PuGRF9* was specifically and highly expressed in the buds at the seedling stage and in the ML and AP at the rapid growth stage. Perhaps it has a similar function to its homologous *AtGRF9* [37], that is, negatively regulating leaf growth. We also noticed that *PuGRF12a* and *PuGRF12b*, which belong to the same clade as *PuGRF9*, have different expression patterns and obvious tissue specificity. Wang et al. identified that the overexpression of *PagGRF12a* and *PagGRF12b* promoted leaf development [3], while *PagGRF12a* interacted with *PagGIF1b* to upregulate *PagXND1a* expression, thereby inhibiting xylem development in *Populus alba × P. glandulosa* [38]. Therefore, we infer that this paralogous pair, *PuGRF12a* and *PuGRF12b*, may compensate for the negative regulatory function similar to *AtGRF9* or represent novel functions evolved in woody plants to influence stem development. Additionally, the overexpression of *PagGRF11* resulted in stem development and dwarfing [39]. The complex mechanisms underlying GRF involvement in woody plant growth and development require further dissection.

The key genes of plants exist in intricate regulatory networks based on their adaptability and evolutionary characteristics. These key genes interact with the environment, generating complex molecular and cellular activity patterns, which in turn induce various plant phenotypes, enabling plants to adapt to variable environments. ABA is an important signaling molecule in plants that regulates physiological processes and helps plants to cope with various stress conditions. It can interact and crosstalk with MeJA, SA, etc., to form a complex signaling network, which regulates the development of plant leaves and roots, affects stomatal movement, and stabilizes the cell’s osmotic capacity to adapt to salt or drought stress [40,41]. Our study preliminarily analyzed the expression patterns of PuGRFs under the stresses of NaCl and 7% PEG6000, as well as under the treatments of ABA, MeJA, and SA. The results indicated that *PuGRF1/2a*, *PuGRF1/2b*, *PuGRF7b*, and *PuGRF10b* were induced in roots under the stresses of NaCl, 7% PEG6000, and ABA treatment, respectively, suggesting that they may be involved in root development-related responses in the ABA-mediated drought-resistance or salt-tolerance pathways. In leaves treated with ABA, MeJA, and SA respectively, 13 genes could be upregulated. Although the expression levels of these genes in leaves under ABA treatment reached their peaks at 24 h or 72 h, most of these genes showed a consistent expression pattern under MeJA and SA treatments. Notably, among these 13 genes, 7 genes (*PuGRF6a*, *PuGRF7b*, *PuGRF8*, *PuGRF10a*, *PuGRF10b*, *PuGRF12a*, and *PuGRF12b*) were induced in leaves under the stresses of NaCl and 7% PEG6000. These results suggest that these genes are crucial for *P. ussuriensis* to cope with environmental changes and are likely to respond to drought or salt stress by regulating stomatal changes. Consistent with this, similar findings have also been observed in studies of other plants. For example, ABA upregulates the expression of *AtMYB41*, *AtMYB74*, and *AtMYB102*, and mediates the AtMYB41-BRM module to regulate the stomatal movement of *A. thaliana*, thereby enhancing its drought tolerance [42]. In the regulatory pathway where *AtERF114/115/109* targets non-canonical JAZ proteins to regulate JA signaling, JA induces the transcription of *AtERF114*. The accumulated *AtERF114/115/109* inhibits the activity of *JAZ8* by forming a positive feedback loop, further promoting JA-induced root hair elongation [43]. In the AtDELLA-GRF module that regulates growth under cold stress, GA regulates the upregulation of GRF genes following cold stress. Moreover, the cold stress-induced accumulation of DELLA positively regulates their expression, and GRF proteins interact with DELLA [19]. These analytical results establish potential connections among woody plants, ABA signal transduction, the drought-stress response, and the MeJA response, which contribute to coordinating the growth, development, and stress resistance of woody plants.

The miR396-GRF module, characterized by relatively conserved functions, has been demonstrated to play pivotal roles across multiple species. It is intricately involved in research related to plant morphogenesis and stress resistance, as detailed in references [22,44]. Moreover, the more complex integrated module miR396-GRF/GIF has been shown to effectively promote plant regeneration and enhance transgenic efficiency, as reported in [45,46,47]. Evolutionary analysis of *GRF* and *GIF* gene families across multiple species reveals that GRF and GIF proteins share similar biological functions, while their interactions are highly conserved [48,49,50]. Given the prediction that all *PtGRFs* are susceptible to recognition and cleavage by miR396, our initial step was to predict the interacting proteins of *PuGRFs*. This was aimed at comprehensively exploring and enriching the potential functions of *PuGRFs*. Through a meticulous analysis of the network interaction relationships among the 19 PuGRFs, we determined that there was no interaction among all members of the PuGRF family. Specifically, eight PuGRFs exhibited a potential connection with PuGIFa/b, while the remaining 11 PuGRFs were more likely to cooccur in specific environmental contexts. Subsequently, we harnessed the HDOCK server to simulate the interaction model between PuGRFs and PuGIFs in *P. ussuriensis*. Intriguingly, we discovered that PuGRF1/2c and PuGRF1/2d, which belong to the same clade and possess highly similar sequences, might have divergent interaction relationships with PuGIFa/b. This finding was corroborated through a series of well-designed experiments. The distinct interaction effects not only reflect the remarkable adaptability of plants to their surroundings, but also underscore the conservation of GRF evolution. On one hand, plants are capable of leveraging existing protein structures and functional modules and can achieve novel functions through subtle modifications. For instance, under particular environmental conditions, such as periods of stress or specific growth stages, PuGRF1/2d, which interacts with PuGIFa/b, exerts its function. Conversely, under other conditions, PuGRF1/2c, which does not interact with PuGIFa/b, becomes active, enabling plants to respond nimbly to diverse environmental challenges. On the other hand, these two PuGRFs with strikingly similar sequences may exhibit functional redundancy, implying that they might still perform their functions, even in the absence of PuGIFa/b. In any event, this divergence merits our close attention. Whether it represents functional specificity or redundancy ultimately necessitates further experimental validation. These findings collectively indicate that the functional roles of GRF proteins are intricately linked to their multifaceted interaction networks, thereby establishing a molecular framework that connects GRF-mediated biological processes with their dynamically evolving protein partnership landscapes.

In summary, our research represents the first in-depth exploration of the GRF gene family in *P. ussuriensis*. Through a comprehensive step-by-step analysis of the evolutionary trends and functional predictions, we found a substantial amount of reliable evidence that indicates that *PuGRFs* are mainly expressed in young tissues during the seedling stage, and there is a significant differentiation in tissue specificity during the rapid growth stage. Additionally, *PuGRFs* mainly respond to hormonal changes in leaves, and they may be mediated by ABA and involved in root development and stomatal movement under drought or salt stress. Further predictive exploration suggested potential interaction relationships between PuGRFs and genes of other domain types, among which eight PuGRFs might interact with PuGIFa/b. Through experiments, we also found that the homologous genes PuGRF1/2c and PuGRF1/2d, despite their highly identical sequences, showed significant differences in their interactions with PuGIFa/b.

## 4. Materials and Methods

### 4.1. Identifcation of PuGRFs in P. ussuriensis

The genomic information of *P. ussuriensis* was derived from a large amount of transcriptional sequencing data within our research group. We downloaded the GRF family proteins of *P. trichocarpa*, *A. thaliana*, and *O. sativa* from Phytozome (https://phytozome-next.jgi.doe.gov/, accessed on 15 April 2024), TAIR (https://www.arabidopsis.org/, accessed on 15 April 2024), and RiceData (http://www.ricedata.cn/gene/, accessed on 15 April 2024). Based on the GRF proteins of known species, we used BLASTP (BLAST: Basic Local Alignment Search Tool, accessed on 15 April 2024) to search for potential *GRF* transcription factors (TFs) in *P. ussuriensis*. Potential GRF transcription factors were identified from the protein sequences of *P. ussuriensis* by HMMER3 (http://www.hmmer.org/, accessed on 15 April 2024). The results of BLASTP and HMMER3 were comprehensively analyzed to select potential *GRFs*. The protein domains of the originally identified members were analyzed using the online software SMART (http://smart.embl.de/, accessed on 15 April 2024) and NCBI-CDD (https://www.ncbi.nlm.nih.gov/cdd/, accessed on 15 April 2024). The physical and chemical properties of *PuGRFs* were analyzed using the online tool ExPASy (SIB Swiss Institute of Bioinformatics | Expasy, https://www.expasy.org/, accessed on 15 April 2024). The subcellular location was predicted by Plant-mPLoc (http://www.csbio.sjtu.edu.cn/bioinf/plant/, accessed on 15 April 2024). The microRNAs were predicted by psRNATarget (psRNATarget: A Plant Small RNA Target Analysis Server (2017 Update), https://www.zhaolab.org/psRNATarget/, accessed on 27 April 2024).

### 4.2. Phylogenetic Investigation of the PuGRFs

The ClustalW method [51] was employed to analyze *P. ussuriensis*, *A. thaliana*, and *O. sativa*. The phylogenetic tree of the *GRF* family was constructed by the neighbor-joining (NJ) method. In MEGA7.0 software [52], 1000 iterations were performed for bootstrap testing, and then the phylogenetic tree was beautified using the online software Evolview (https://evolgenius.info/evolview, accessed on 6 May 2024).

### 4.3. Analysis of Conserved Motifs, Gene Structure, and Promoter Regions in PuGRFs

The conserved motifs for PuGRF proteins were identified using MEME (https://meme-suite.org/meme/tools/meme, accessed on 20 May 2024) [53]. The parameters were set to 20 motifs while keeping other parameters at their default values. Detailed information about 20 conserved motifs is listed in the Supplementary Table S4. The gene structure was visualized using TBtools (version 2.202, South China Agricultural University, China) [54]. The promoters of the *PuGRFs* were obtained from the genome of *P. ussuriensis*. The promoter region, specifically the 3 kb sequences upstream of the translation initiation site (ATG), was analyzed by the online software PlantCARE (http://bioinformatics.psb.ugent.be/webtools/plantcare/html/, accessed on 17 April 2024) [55] to identify the cis-acting elements. The conserved motifs and cis-elements of the *PuGRFs* were visualized with TBtools.

### 4.4. Plant Materials and Treatments

The plant material was sourced from the wild-type *P. ussuriensis* at Northeast Forestry University. It was sterilized with 0.1% HgCl_2_ solution and then cultured in woody plant medium (WPM) supplemented with 20 g/L sucrose and 6 g of agar using tissue culture technology [56,57]. The tissue culture chamber was maintained at 25 °C with a photoperiod of 16 h of light and 8 h of darkness and a light intensity of 46 µmol m^−2^ s^−1^. Uniformly grown, healthy three-week-old in vitro plants (seedling stage) obtained from the above culture protocols were used for abiotic stress treatments (21 plant samples per treatment, with 3 plants per time point), phytohormone treatment (21 plant samples per treatment, with 3 plants per time point), and tissue-specific analysis (different tissues from 40 plant samples were pooled separately). Additionally, uniformly grown, healthy two-month-old soil-grown seedlings (rapid growth stage) were used for tissue-specific analysis (different tissues from 12 plant samples were pooled separately). For abiotic stress and phytohormone treatments, plants were exposed to a WPM medium containing 200 mM NaCl, 7% PEG6000, 200 µM ABA, 100 µM MeJA, and 100 µM SA. The concentrations of NaCl, PEG6000, ABA, SA, and MeJA were based on repeated laboratory trials and slightly adjusted [58,59,60,61]. Each treatment lasted for 0 h, 3 h, 6 h, 12 h, 24 h, 72 h, and 7 d. Three biological replicates were conducted for each treatment, and roots and leaves at different time points need to be quickly frozen with liquid nitrogen at −80 °C for later use.

### 4.5. RNA Isolation and RT-qPCR Analysis

Highly purified RNA was extracted by employing the Universal Plant Total RNA Isolation Kit (Spin-Column, BioTeke, Beijing China). The MonScript™ RTIII All-in-One Mix with dsDNase (monadbiotech, Suzhou, China) was utilized to obtain cDNA, with the following reaction system: 1000 ng of total RNA, 4 µL of MonScript™ 5×RTIII All-in-One Mix, 1 µL of MonScript™ dsDNase, and an appropriate amount of nuclease-free water to bring the total volume to 20 µL. The cDNA was diluted 10-fold (cDNA: nuclease-free water = 1:10) for further RT-qPCR analysis. The RT-qPCR specific primers were designed by the NCBI Primer Blast tool (https://www.ncbi.nlm.nih.gov/tools/primer-blast/index.cgi, accessed on 31 May 2024), and the β-Actin gene (EF418792.1) was selected as an internal reference. TransStart^®^ Top Green qPCR Super Mix (TransGen-AQ131, Beijing, China) was used to conduct RT-qPCR, with the following reaction system: 10 µL of 2 × TransStart^®^ Top Green qPCR Super Mix, 6 µL of double-distilled H_2_O, 2 µL of diluted template, and 1 µL each of forward primer and reverse primer. The qTOWER 3G Cycler and qPCR software (version 3.2, Analytik Jena, Jena, Germany) were used as a work program, and the 2^−∆∆CT^ method [62] was employed to perform the relative gene expression level analysis. Triplicate independent technical and biological replications were used in the analysis. The primers for RT-qPCR are listed in Supplementary Table S1. SPSS software (version 20, IBM, Chicago, IL, USA) was used to analyze the data by performing one-way analysis of variance (ANOVA) to assess significant differences between the control and each treatment. Significance was defined as * *p* < 0.05 and ** *p* < 0.01.

### 4.6. Protein-Protein Interaction Studies of PuGRFs

To analyze the protein-protein interactions (PPIs), the STRING 12.0 database [63] (https://string-db.org/, accessed on 8 May 2024) was employed, utilizing the default parameters and Populus trichocarpa as the reference organism. HDOCK SERVER [20,64,65,66,67,68] (http://hdock.phys.hust.edu.cn/, accessed on 8 May 2024) was used to simulate Protein-protein docking.

### 4.7. Y2H Assays

The Y2H assay was carried out using the Matchmaker Gold Yeast Two-Hybrid System (Coolaber-YH2011, Beijing, China). To detect the interactions between PuGRF1/2c, PuGRF1/2d, and two PuGIF proteins (PuGIFa and PuGIFb), the coding sequences (CDS) of *PuGRF1/2c* and *PuGRF1/2d* were amplified from complementary DNA (cDNA) and cloned into the pGBKT7 vector. Meanwhile, the full-length coding sequences of *PuGIFa* and *PuGIFb* were cloned into the pGADT7-rec_2_ vector. To identify the domains of PuGRF1/2c and PuGRF1/2d involved in the interaction, the coding sequences of *PuGRF1/2c* and *PuGRF1/2d* and their derivative fragments were cloned into the pGBKT7 vector. The primers used for plasmid construction are listed in Supplementary Tables S2 and S3. Then, they were transformed into the yeast (*Saccharomyces cerevisiae*) strain Y2H Gold respectively. The transformed yeast cell suspensions were spread onto the plates of SD/-Trp, SD/-Trp/-His/-Ade, and SD/-Trp + X-a-gal medium. PuGRF1/2c^148−259aa^, PuGRF1/2d^150−261aa^, and the two PuGIFs were co-transformed into the yeast strain Y2H Gold respectively. Positive (BD-53 + AD-T) and negative (BD-lam + AD-T) controls were included. The transformed yeast cell suspensions were spread onto the plates of SD/-Trp/-Leu (-WL), SD/-Trp/-Leu/-His(-WLH), and SD/-Trp/-Leu/-His+ X-a-ga (-WLH+ X-a-gal) medium to detect protein–protein interactions. All the plates were incubated at 30 °C for 72 h and experiments were repeated three times.

### 4.8. BiFC

For BiFC assays, the full-length coding sequences of *PuGRF1/2d*, *PuGIFa*, and *PuGIFb* were inserted into the pENTER/D-TOPO vector respectively. Subsequently, they were recombined with nEYFP/pUGW2 and cEYFP/pUGW2 using Gateway recombination technology, respectively. According to the established protocol [69], each pair of constructs was co-transfected into poplar protoplasts together with the nuclear localization marker H2A–1:mCherry. The empty vectors of yellow fluorescent protein C-terminus (YFPC) and yellow fluorescent protein N-terminus (YFPN) were used as a negative control. A confocal laser-scanning microscope (LSM 800; Zeiss, Oberkochen, Germany)was employed to detect the fluorescence signals. Three biological replicates were tested for both the interaction groups and the negative control groups. The primers used for plasmid construction are listed in the Supplementary Tables S2 and S3.

### 4.9. LCI Assays

The LCI assays were performed in *N. benthamiana* leaves according to the method described previously [70]. The full-length *PuGRF1/2d* was cloned into the pCAMBIA1300-nLUC vector, while the full-length *PuGIFa* and *PuGIFb* were cloned into the pCAMBIA1300-cLUC vector. The primers used for plasmid construction are listed in Supplementary Tables S2 and S3. The resulting constructs were separately introduced into *Agrobacterium tumefaciens* strain GV3101(p19). The transformed cells were cultured in LB liquid medium containing 2-(N-morpholino) ethanesulfonic acid (Sigma, Darmstadt, Germany) and acetosyringone (Sigma, Germany) at 28 °C. After culturing at 28 °C for 16 h, the bacterial cells were harvested and resuspended in sterile water containing MgCl_2_ and acetosyringone to an optical density (OD_600_) of 1.5. Equal volumes of the two bacterial suspensions carrying the specific constructs were mixed, incubated at room temperature for 3 h, and then injected into the leaves of *N. benthamiana*. The plants with infiltrated leaves were cultured at 23 °C under a 16 h light/8 h dark photoperiod for 3–4 days. Then, 1 mM luciferin was sprayed onto the infiltrated leaves, which were left in the dark at 37 degrees Celsius for 10 min, and a CCD imaging device was used to capture LUC images.

### 4.10. In Vitro Pull-Down Assays

The in vitro pull-down assay was carried out with finetuning according to the previously described method [71]. An amount of 200 μL of MBP-PuGRF1/2d was mixed with equal amounts of GST-PuGIFa and GST-PuGIFb and then added to 50 μL of glutathione magnetic beads (PureCube Glutathione MagBeads32201, Cube Biotech, Monheim, Germany) equilibrated with BW buffer (50 mM NaH_2_PO_4_, 150 mM NaCl) in a 2 mL centrifuge tube. The mixture was incubated at 4 °C for two hours. Beads were washed four times, and proteins were eluted with 40 μL elution buffer (125 mM Tris base, 150 mM NaCl, 0.1% (*v*/*v*) Triton X-100, 50 mM Reduced glutathione, and 1 mM DTT). Eluates were resolved by SDS-PAGE and immunoblotted using anti-GST (1:3000, Abclonal-AE006, ABclonal Technology, Wuhan, China) and anti-MBP (1:3000, Abclonal-AE016, ABclonal Technology, Wuhan, China) antibodies. The primers used for plasmid construction are listed in Supplementary Tables S2 and S3.

## 5. Conclusions

In this study, we identified 19 *PuGRFs* in *P. ussuriensis* and characterized their physicochemical properties, evolutionary relationships, and dynamic expression profiles. Phylogenetic analysis classified these genes into six distinct clades, revealing lineage-specific structural conservation and diversification. Notably, *PuGRFs* exhibited pronounced tissue-specific expression patterns, predominantly in young tissues during seedling stages and diversified expression across mature tissues during rapid growth phases. Furthermore, these genes demonstrated robust responsiveness to abiotic stresses (salt and osmotic) and phytohormones (ABA, MeJA, and SA), with distinct temporal and spatial regulatory dynamics. Protein interaction predictions and experimental validations revealed functional divergence among closely related *PuGRFs*, suggesting evolutionary fine-tuning and potential redundancy. This duality highlights the adaptability of *GRFs* in balancing conserved roles and lineage-specific innovations. Our findings not only provide the first genome-wide framework for the *GRF* family in *P. ussuriensis*, but also uncover critical insights into their roles in coordinating growth, stress responses, and hormonal signaling.

## Figures and Tables

**Table 1:** Physicochemical properties of GRFs in *Populus ussuriensis*.

Gene Name	Gene ID of the Homologous Genes in *P. trichocarpa*	Full (bp)	Protein Length (aa)	Theoretical (pI)	Molecular Weight (Da)	Predicted Location(s)	Predicted microRNAs (ptc-miR)
*PuGRF1/2a*	Potri.007G007100	1833	611	7.28	66,518.92	Nucleus	396a-396g, 7822, 159c, 169y, 6439a, 6454, 7835
*PuGRF1/2b*	Potri.014G007200	1824	607	6.44	65,962.14	Nucleus	396a-396g, 6421, 7829
*PuGRF1/2c*	Potri.002G115100	1818	605	7.22	65,496.67	Nucleus	396a-396g, 169b, 164a-164f, 399d, 6426a-6426b
*PuGRF1/2d*	Potri.014G012800	1836	611	8.61	66,179.14	Nucleus	396a-396g, 164a-164f, 397a, 6444, 6466, 6473
*PuGRF3/4*	Potri.006G115200	1203	400	7.33	43,882.40	Nucleus	396a-396g, 390a-d, 482d, 7813, 7824
*PuGRF5a*	Potri.003G065000	1023	340	7.25	37,883.77	Nucleus	396a-396g, 2111a-2111b, 399a-399d, 399j, 478e, 395b-395k, 475d
*PuGRF5b*	Potri.001G169100	1047	348	7.74	39,163.48	Nucleus	6426a-6426b, 396a-396g, 399f-399i, 399d, 6444, 159c, 169n, 393a-363c, 481d
*PuGRF6a*	Potri.006G143200	1287	428	8.88	48,392.25	Nucleus	396a-396g, 476b, 7814, 156k-156l, 395a, 169s, 172a-172f, 390a-390d, 476a, 6442
*PuGRF6b*	Potri.018G065400	1155	384	8.64	43,491.38	Nucleus	396a-396g, 156a-156l, 390a-390d, 7814, 156a-156f, 474a-474b, 7817b
*PuGRF7a*	Potri.012G022600	1608	535	7.71	58,256.64	Nucleus	396a-396g, 172b, 172d-172e, 172g-172i, 475a-475b, 475d, 1444a, 6461, 6475, 7835, 7841
*PuGRF7b*	Potri.015G006200	1614	537	8.37	68,981.47	Nucleus	396a-396g, 1444a-1444c, 172a-172i, 6425a-6425e, 6447
*PuGRF8*	Potri.001G082700	1572	523	6.96	56,896.02	Nucleus	396a-396g, 472b, 482d, 390a-390d, 6471
*PuGRF9*	Potri.014G071800	1377	458	9.11	50,349.58	Nucleus	396a-396g, 6470, 6427
*PuGRF10a*	Potri.001G132600	1086	361	9.3	40,379.25	Nucleus	396a-396g, 172b, 172g, 6445a-6445b, 6472, 7828
*PuGRF10b*	Potri.003G100800	1011	336	9.1	37,297.52	Nucleus	396a-396g, 6445a-6445b, 171l, 7815
*PuGRF11a*	Potri.013G077500	1023	340	7.72	37,402.25	Nucleus	396a-396g, 164a-164f, 475d, 7822
*PuGRF11b*	Potri.019G042300	987	328	8.96	36,233.07	Nucleus	396a-396g, 390a-390d, 408, 164f, 6423, 7813
*PuGRF12a*	Potri.001G114000	609	202	10.06	22,477.85	Nucleus	396a-396g
*PuGRF12b*	Potri.003G118100	606	201	9.94	22,313.44	Mitochondrion; Nucleus	396a-396g, 6480

**Table 2:** The scores of the HDOCK SERVER prediction model.

Name		Model 1		Model 2		Model 3		Model 4		Model 5	
		Dock	Confidence	Dock	Confidence	Dock	Confidence	Dock	Confidence	Dock	Confidence
PuGRF1/2a	PuGIFa	−171.37	0.6053	−161.52	0.5573	−160.81	0.5538	−157	0.5349	−154.84	0.5242
	PuGIFb	−185.91	0.6722	−164.85	0.5737	−160.97	0.5546	−160.28	0.5512	−154.92	0.5246
PuGRF1/2b	PuGIFa	−235.84	0.8477	−234.64	0.8446	−209.29	0.766	−193.59	0.7051	−190.97	0.6941
	PuGIFb	−203.77	0.7456	−195.5	0.713	−190.32	0.6913	−183.81	0.6629	−181.4	0.652
PuGRF1/2c	PuGIFa	−144.31	0.4716	−138.13	0.4409	−136.26	0.4317	−129.22	0.3976	−127.87	0.3911
	PuGIFb	−145.95	0.4798	−134.19	0.4216	−131.63	0.4092	−130.46	0.4035	−128.24	0.3929
PuGRF1/2d	PuGIFa	−190.73	0.6931	−177.09	0.6322	−171.51	0.6059	−170.98	0.6034	−170.94	0.6032
	PuGIFb	−184.81	0.6673	−164.6	0.5725	−159.5	0.5474	−157.81	0.539	−156.01	0.53
PuGRF3/4	PuGIFa	−196.47	0.717	−194.87	0.7104	−187.22	0.678	−185.67	0.6712	−184.51	0.666
	PuGIFb	−191.07	0.6945	−185.84	0.6719	−180.97	0.6501	−179.17	0.6418	−166.01	0.5794
PuGRF5a	PuGIFa	−189.6	0.6886	−185.4	0.67	−173.42	0.615	−170.67	0.6019	−170.67	0.6019
	PuGIFb	−202.75	0.7417	−189.25	0.6868	−179.49	0.6433	−178.57	0.6391	−177.39	0.6336
PuGRF5b	PuGIFa	−176.72	0.6305	−174.26	0.619	−167.51	0.5867	−165.87	0.5787	−165.76	0.5782
	PuGIFb	−190.4	0.6917	−177.21	0.6328	−176.9	0.6313	−176.57	0.6298	−176.51	0.6295
PuGRF6b	PuGIFa	−248.48	0.8776	−218.49	0.7973	−212.27	0.7765	−209.91	0.7682	−204.96	0.7501
	PuGIFb	−203.15	0.7433	−202.32	0.7401	−200.22	0.7319	−196.61	0.7175	−195.06	0.7112

**Note:** The docking scores are calculated by the knowledge-based iterative scoring function ITScorePP or ITScorePR. A more negative docking score means a more possible binding model. Confidence score = 1.0/[1.0 + e^0.02∗(Docking_Score+150)^]. The docking score-dependent confidence score to indicate the binding likeliness of two molecules is as follows: confidence score > 0.7, the two molecules would be very likely to bind; 0.5 < confidence score < 0.7, the two molecules would be possible to bind; confidence score < 0.5, the two molecules would be unlikely to bind.

## Data Availability

I have shared the code to my data in the manuscript.

## Supplementary Materials

The following supporting information can be downloaded at: https://www.mdpi.com/article/10.3390/ijms26073288/s1.
