# Supplementary material for: Genome-Wide Identification and Characterization of the Growth-Regulating Factor Gene Family Responsive to Abiotic Stresses and Phytohormone Treatments in Populus ussuriensis
Source: Int J Mol Sci. 2025 Apr 1;26(7):3288. doi: 10.3390/ijms26073288 (PMC11990005; doi:10.3390/ijms26073288)
Supplement: Supplementary file 1 [file ijms-26-03288-s001.zip › ijms-3509460-supplementary.pdf]

Supplementary Table S1. The primers of *PuGRFs* for RT-qPCR.

Name	Primer sequence
DL.GRF1/2a-F	TCTTGGTTTGGAGGGTCTTG
DL.GRF1/2a-R	CCTGAGCAGATGCAGATGAA
DL.GRF1/2b-F	GTCGTCGGACTGATGGGAAG
DL.GRF1/2b-R	AGGCACCACCACTGGTTATT
DL.GRF1/2c-F	TCTTGGTTTGGAGGGTCTTG
DL.GRF1/2c-R	CCTGAGCAGATGCAGATGAA
DL.GRF1/2d-F	GCAGAAGTACGGATCTGGGT
DL.GRF1/2d-R	GCAACATGGCATTTGGGGAGC
DL.GRF3/4-F	GTCTCAGGACCTCACTGACCAGGA
DL.GRF3/4-R	CAATACTACCACCTCCACCTCCAC
DL.GRF5a-F	GCTCCTCCAACCAATCAAG
DL.GRF5a-R	TGGCTGCTCTTCCCCAATACC
DL.GRF5b-F	TGGAATAAGGGAGTGTATGGACG
DL.GRF5b-R	TGGCTTCTGGGTTTCAGGT1
DL.GRF6a-F	AGCACTGCTTTGTTTTGGGC
DL.GRF6a-R	TGGCCAAGAGTAAACTTCTCTCA
DL.GRF6b-F	TCCCCATCCCTCCTGATCTC
DL.GRF6b-R	AGACAGTTGGTGGCGATGT
DL.GRF7a-F	TGATGAATGGCCACCAAAGGA
DL.GRF7a-R	ACAAGAAGTATTTGTCCGGTTCG
DL.GRF7b-F	AGGCAATATGGCAGCGTCTT
DL.GRF7b-R	GCCACATCTCTTGAGCACCT
DL.GRF8-F	CAAATGCACAATGGAACGAG
DL.GRF8-R	TGAACCTCACATCCAGTCCA
DL.GRF9-F	CCTGGCACTGGAAGTGAAACT
DL.GRF9-R	TGGTTTTTGTTGACATTGGGGC
DL.GRF10a-F	GCCATATGCATAGAGGCCGT
DL.GRF10a-R	TGCAACTTGTGGTGCAAGTG
DL.GRF10b-F	ACCGTTCAAGAAAGCCTGTG
DL.GRF10b-R	AGCCTAAGGGCCCAGAATTA
DL.GRF11a-F	GGCGGTGGGATCAAGATCAC
DL.GRF11a-R	TGATGGGTGCAAGAAACCAG
DL.GRF11b-F	AGCTTCCAGAACCTTCCATTGCAC
DL.GRF11b-R	TGCATTGTCCAGCTGTGAGTCTAT
DL.GRF12a-F	AACAGGGGTCTCCAGAAGGA
DL.GRF12a-R	TTGAGGCCAGACCAAGTGTC
DL.GRF12b-F	CATGGAGGGAAATAGGCGCA
DL.GRF12b-R	AGACCACCAAGGGAAGCAGT
Actin-F	TGTTGCCCTTGACTATGAGCAGGA
Actin-R	ACGGAATCTCTCAGCTCCAATGGT

Supplementary Table S2. The primers of *PuGRF1/2c* and *PuGRF1/2d* for vector construction.

Name	Primer sequence
GRF1/2c_clone-F	ATGGATTTTGGGGTTCAGGTGG
GRF1/2c_clone-R	TTACAAGGCAGGCAATGATGAAGA
GRF1/2d_clone-F	ATGGATTTTGGGGTTCAGGTGGG
GRF1/2d_clone-R	TTACATGGCAGGCAATGAAGAAGA
GRF1/2c_BD-F	ATGGCCATGAGGCGAATT CCCGGGATGGATTTTGGGGTT
GRF1/2c_BD-R	GCTGCAGGTCGACGGATCCCCGGGCAAGGCAGGCAATGA
GRF1/2d_BD-F	ATGGCCATGAGGCGAATTCCCGGGATGGATTTTGGGGTTCAG
GRF1/2d_BD-R	GCTGCAGGTCGACGGATCCCCGGGCATGGCAGGCAATGA
GRF1/2c-C_BD-F	ATGGCCATGAGGCGAATTCCCGGGATGGAAGGACAATCAGGCCAT
GRF1/2c-C_BD-R	GCTGCAGGTCGACGGATCCCCGGGCAAGGCAGGCAATGATGA
GRF1/2d-C_BD-F	ATGGCCATGAGGCGAATTCCCGGGATGGAAGGCCAATCAGGC
GRF1/2d-C_BD-R	GCTGCAGGTCGACGGATCCCCGGGCATGGCAGGCAATGAAGA
GRF1/2c-148-259aa_BD-F	ATGGCCATGAGGCGAATTCCCGGGATGCCATTCACTCAATTAC
GRF1/2c-148-259aa_BD-R	GCTGCAGGTCGACGGATCCCCGGGCACAGGCTTTCTTGA
GRF1/2d-150-261aa_BD-F	ATGGCCATGAGGCGAATTCCCGGGATGCCATTCACTCAATCACAA
GRF1/2d-150-261aa_BD-R	GCTGCAGGTCGACGGATCCCCGGGCACAGGCTTTCTTGAAC
GRF1/2c-N_BD-F	ATGGCCATGAGGCGAATT CCCGGGATGGATTTTGGGGTT
GRF1/2c-N_BD-R	GCTGCAGGTCGACGGATCCCCGGGCCATCTAGTCTCAGTC
GRF1/2d-N_BD-F	ATGGCCATGAGGCGAATTCCCGGGATGGATTTTGGGGTTCAG
GRF1/2d-N_BD-R	GCTGCAGGTCGACGGATCCCCGGGCCATCTGGTCTCGGTCAA
GRF1/2d_ attl-F	GTACAAAAAAGCAGGCTCAGGGGATATCATGGATTTTGGGGTTCAG
GRF1/2d_ attl-R	AAGAAAGCTGGGTGCAGGGCGATATCCATGGCAGGCAATGA
GRF1/2d_nLUC-F	ACGGGGGACGAGCTCGGTACCATGGATTTTGGGGTTCAGGT
GRF1/2d_nLUC-R	CGCGTACGAGATCTGGTCGACCATGGCAGGCAATGAA
GRF1/2d_MBP-F	TCCATGGGCGGCCGCGAT ATCATGGATTTTGGGGTTCAG
GRF1/2d_MBP-R	GAATTCGGATCCGTCGACGAT ATCCATGGCAGGCAATGA

Supplementary Table S3. The primers of *PuGIFa* and *PuGIFb* for vector construction.

Name	Primer sequence
GIFa_clone-F	ATGCAACAGCACCTGATGCAGAT
GIFa_clone-R	TCAATTCCCATCATCTGCAGATTTCAAGTA
GIFb_clone-F	ATGCAACAGCACCTGATGCAGATGC
GIFb_clone-R	TCAGTTCCCATCATCAGCAGATTTCAAG
GIFa_AD-F	GCAGAGTGG CCATTATGGCCCGGGATGCAACAGCACCTG
GIFa_AD-R	GCGGCCGACATGTTTTTTCCCGGGAATTCCCATCATCTGCA
GIFb_AD-F	GCA GAG TGG CCA TTA TGG CCCGGGATGCAACAGCACCTG
GIFb_AD-R	GCGGCCGACATGTTTTTTCCCGGGGTTCCCATCATCAGCAG
GIFa_GST-F	CGTGGATCCCCGGAATTCCCGGGATGCAACAGCACCTG
GIFa_GST-R	CTCGAGTCGACCCGGGATTCCCATCATCTGCA
GIFb_GST-F	CGTGGATCCCCGGAATTCCCGGGATGCAACAGCACCTG
GIFb_GST-R	CTCGAGTCGACCCGGGGTTCCCATCATCAGCAG
GIFa_attl-F	GTACAAAAAAGCAGGCTCAGGGGATATCATGCAACAGCACCTG
GIFa_attl-R	AAGAAAGCTGGGTGCAGGGCGATATC ATTCCCATCATCTGCA
GIFb_attl-F	GTACAAAAAAGCAGGCTCAGGGGATATCATGCAACAGCACCTG
GIFb_attl-R	AAGAAAGCTGGGTGCAGGGCGATATCGTTCCCATCATCAGCAG
GIFa_cLUC-F	TACGCGTCCCGGGGCGGTACCATGCAACAGCACCTG
GIFa_cLUC-R	ACGAAAGCTCTGCAGGTCGACTCAATTCCCATCATCTGC
GIFb_cLUC-F	TACGCGTCCCGGGGCGGTACC ATGCAACAGCACCTG
GIFb_cLUC-R	ACGAAAGCTCTGCAGGTCGACTCAGTTCCCATCATCAGCA

Supplementary Table S4. Detailed information about 20 conserved motifs of 19 *PuGRFs*.

Motif	Motif Consensus
motif 1	TDPEPGRCRRTDGKKWRCSKDAVPDQKYCERHMHRGRNRSRKPVELQTTT
motif 2	NRFPFTASQWQELEHQALIYKYMVAGVPVPPDLLJPIKKSL
motif 3	QQSLRQFFDEWPKDQ
motif 4	KDYRYLHGLKEEVDEHAFLPEALGSTRGLQDSST
motif 5	DANEPNQKQANWIPISWGTSIGGPLGEVLHTSNNNA
motif 6	DRTQLSISIPMAPADFSSSSSSPNNEKJALSPLRLSREFDPIQMGLGV
motif 7	WGCFQMGYGRN
motif 8	IPKQDNQYPQLLQTEFGLVTSDSLLNPSQ
motif 9	LREGWDGSPRJGSSPTGVLQKSAFASLSN
motif 10	HKGYSYLQFQSFADHDKPEQQKPGQHNYLLGTDIIKSSATRSIKLEK
motif 11	HDQFFGPMPQPYIQTPVFVNKTSEKIATFDVNGAFGSTFKEPRSFDWMLK
motif 12	PGFLHHPNLSYCSYYGKK
motif 13	QDDYWRASKMPKNDDFSVTKTMPLHQPAPLLRPNYMFSDDSRQQEHMLSF
motif 14	ERNHTALSFPYFQLTSSAYNRNAGYNSGILNA
motif 15	TWHLTSQVPANPVPESRNGALLQNYPQLRTLQDFEPLTVDDAPSKQQZZQ
motif 16	ERPQDASAJSMLSSTIDRKSDESPF
motif 17	TTQLSISIPMAPNFLPTSYHSPNB
motif 18	QLGLDGLVGPDTSNSGPAHLASPDPEPKQ
motif 19	KKNNENPSLCEDVPGSIIIS
motif 20	VDFGVKLHQPIDHHQSFP

Supplementary Table S5. Detailed information about 31 *cis*-elements of 19 *PuGRFs*.

cis-element	Sequence	Funcation	
ARE	AAACCA	*cis*-acting regulatory element essential for the anaerobic induction	
Box III	atCATTTTCACt	Pisum sativum	protein binding site
Pc-CMA2a	CAGCCAATCACAG	Pisum sativum	part of a light responsive element
AT-rich	TAAAATACT	Pisum sativum	element for maximal elicitor-mediated activation (2copies)
CAT-box	GCCACT	*cis*-acting regulatory element related to meristem expression	
TGA-element	AACGAC	auxin-responsive element	
ABRE	ACGTG	*cis*-acting element involved in the abscisic acid responsiveness	
LTR	CCGAAA	*cis*-acting element involved in low-temperature responsiveness	
CGTCA-motif	CGTCA	*cis*-acting regulatory element involved in the MeJA-responsiveness	
TC-rich repeats	ATTCTCTAAC	*cis*-acting element involved in defense and stress responsiveness	
TGACG-motif	TGACG	*cis*-acting regulatory element involved in the MeJA-responsiveness	
O2-site	GATGATGTGG	*cis*-acting regulatory element involved in zein metabolism regulation	
MRE	AACCTAA	MYB binding site involved in light responsiveness	
GARE-motif	TCTGTTG	gibberellin-responsive element	
MBS	CAACTG	MYB binding site involved in drought-inducibility	
AT-rich element	ATAGAAATCAA	binding site of AT-rich DNA binding protein (ATBP-1)	
RY-element	CATGCATG	*cis*-acting regulatory element involved in seed-specific regulation	
TCA-element	CCATCTTTTT	*cis*-acting element involved in salicylic acid responsiveness	
CCAAT-box	CAACGG	MYBHv1 binding site	
GCN4_motif	TGAGTCA	*cis*-regulatory element involved in endosperm expression	
HD-Zip 3	GTAAT(G/C)ATTAC	protein binding site	
circadian	CAAAGATATC	*cis*-acting regulatory element involved in circadian control	
GC-motif	CCCCCG	enhancer-like element involved in anoxic specific inducibility	
MBSI	TTTTTACGGTTA	MYB binding site involved in flavonoid biosynthetic genes regulation	
P-box	CCTTTTG	gibberellin-responsive element	
SARE	TTCGACCATCTT	*cis*-acting element involved in salicylic acid responsiveness	
AACA_motif	TAACAAACTCCA	involved in endosperm-specific negative expression	
AuxRR-core	GGTCCAT	*cis*-acting regulatory element involved in auxin responsiveness	
A-box	CCGTCC	*cis*-acting regulatory element	
TATC-box	TATCCCA	*cis*-acting element involved in gibberellin-responsiveness	
3-AF3 binding site	CACTATCTAAC	part of a conserved DNA module array (CMA3)	

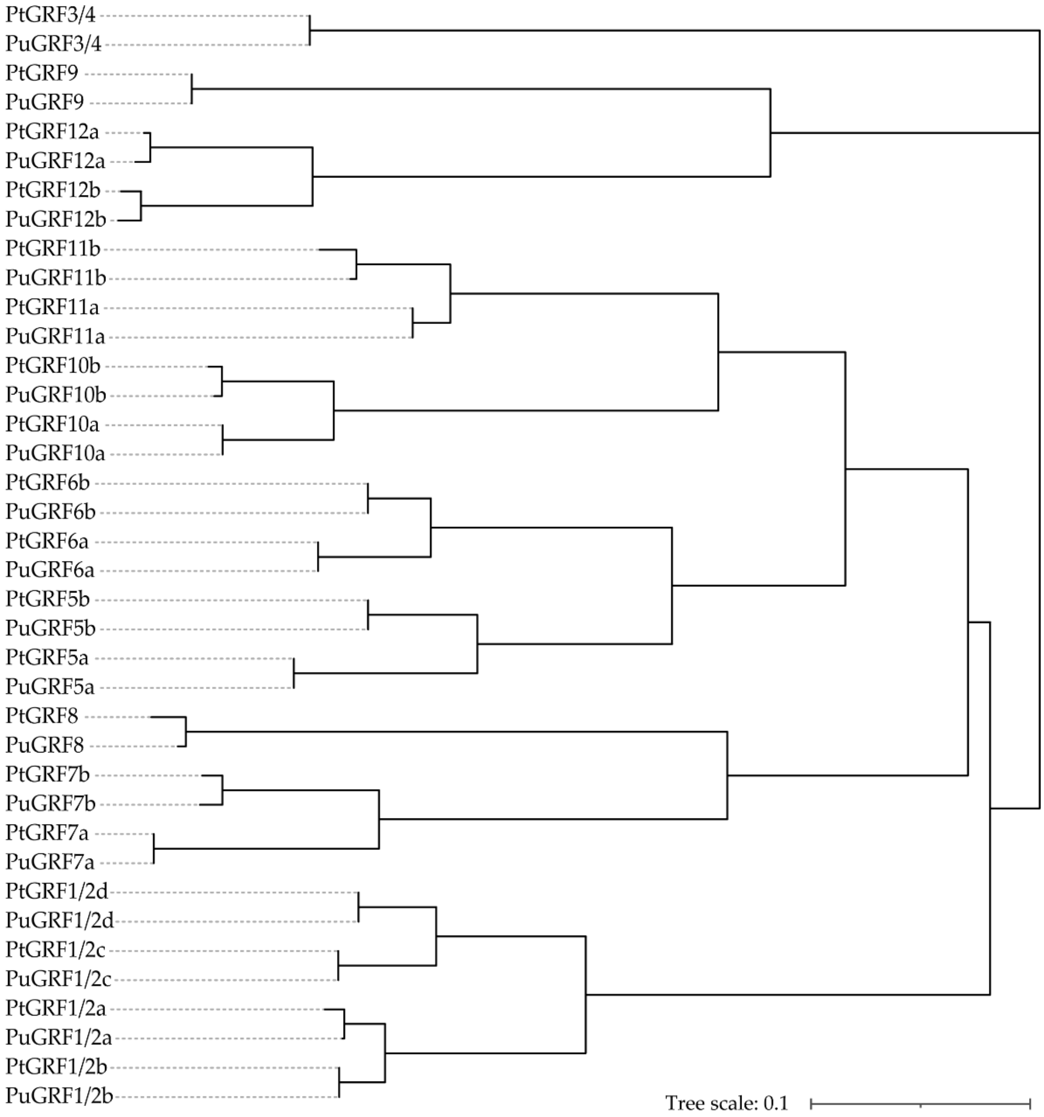

Figure. S1 The phylogenetic tree of PtGRFs and PuGRFs.

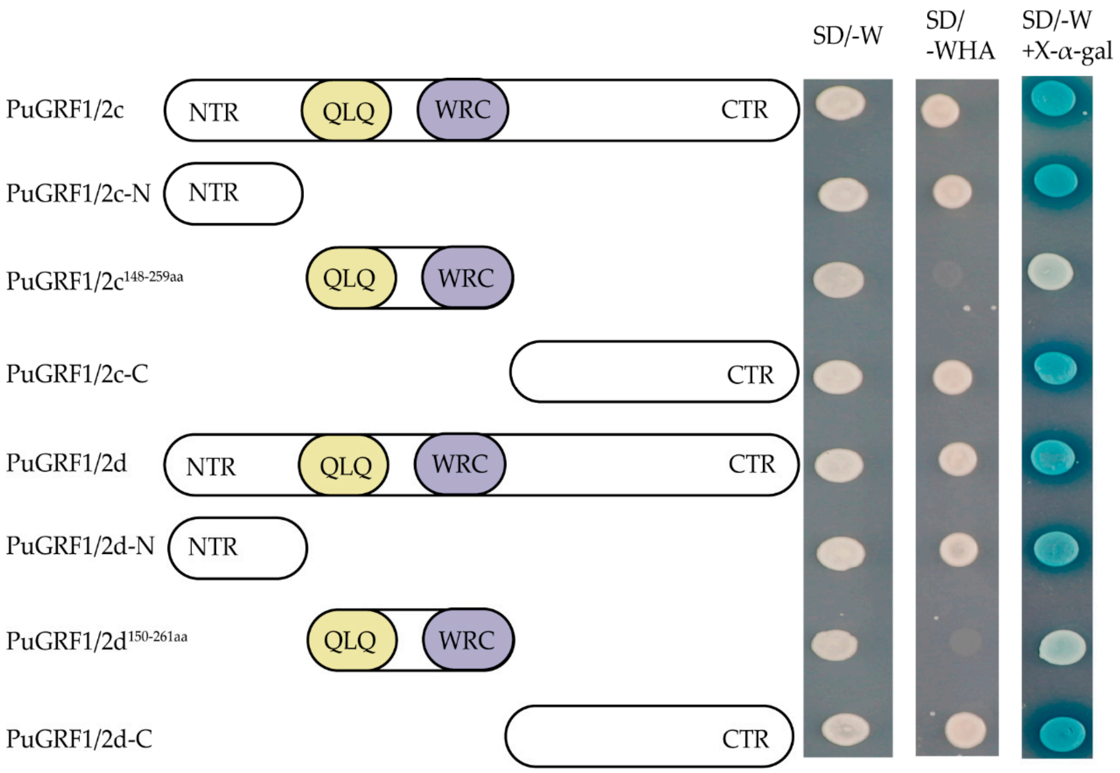

Figure. S2 Detection of the activation activities of PuGRF1/2c and PuGRF1/2d.
SD/-W represents SD/-Trp, SD/-WHA represents SD/-Trp/-His/-Ade, and SD/-W + X-a-gal represents SD/-Trp + X-a-gal.
PuGRF1/2c-N: 1-147aa, PuGRF1/2c-C: 260-606aa. PuGRF1/2d-N: 1-149aa, PuGRF1/2d-C: 262-612aa.

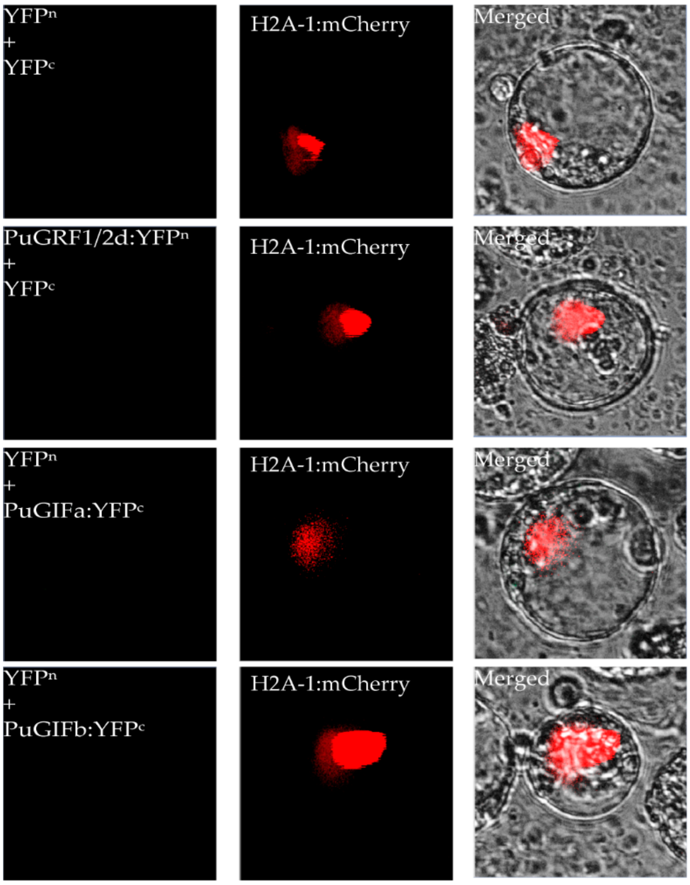

Figure. S3 Negative control for the BiFC verification of the interaction between PuGRF1/2d and PuGIFa/b
